# Clinical Efficacy of Revascularization Surgery for Moyamoya Angiopathy: Long‐Term Results of a European Cohort

**DOI:** 10.1111/ene.70664

**Published:** 2026-06-12

**Authors:** Robert Mertens, Ibrahim Efe, Friedrich Mrosk, Kristin Lucia, Mohammad Asif Iqbal, Kerstin Rubarth, Lukas Mödl, Erin Dirk Sprünken, Lucius Fekonja, Güliz Acker, Peter Vajkoczy

**Affiliations:** ^1^ Department of Neurosurgery Charité – Universitätsmedizin Berlin Berlin Germany; ^2^ Department of Neurosurgery University Hospital Düsseldorf Düsseldorf Germany; ^3^ Department of Neurosurgery University Hospital Frankfurt Frankfurt am Main Germany; ^4^ Institute of Biometry and Clinical Epidemiology Charité – Universitätsmedizin Berlin Berlin Germany

**Keywords:** bypass surgery, Moyamoya angiopathy, Moyamoya disease, Moyamoya syndrome, revascularization surgery, stroke

## Abstract

**Background:**

Moyamoya angiopathy (MMA) is a cerebrovascular disease treated by surgical revascularization. This study aimed to present long‐term outcomes of European MMA patients after surgical revascularization.

**Methods:**

MMA patients treated surgically between 1998 to 2023 were included. Follow‐up comprised clinical and radiological examinations and electronic questionnaires. Patient data were analyzed retrospectively, and logistic regressions were performed to identify predictors of unfavorable outcomes.

**Results:**

A total of 276 patients with 428 surgically treated hemispheres were included. The mean age was 35.0 ± 17.2 years, including 192 women and 84 men, predominantly of Caucasian ethnicity (88.0%). Ischemia (82.2%) and hemorrhage (10.1%) were the most common symptoms at onset. Most adults (80.9%) were treated with combined bypass surgery via mini‐craniotomy. Within 30 days, complications occurred in 6.3% of surgeries, and the perioperative stroke risk was 2.6%. A total of 148 patients underwent in‐house follow‐up. No mRS scores ≥ 5 were observed, with 86.3% of patients achieving a score of 0–2, alongside a bypass patency rate of 95.7% and a stroke risk of 0.3% per patient‐year. Preoperative stroke and silent ischemic MRI lesions were identified as predictors of an unfavorable outcome. Additionally, 201 patients were followed up electronically. No mRS scores ≥ 5 were observed, with 87.9% of patients achieving a score of 0–2. Moreover, 90.7% were independent in daily life, but only 50.5% returned to work or school.

**Conclusions:**

Combined bypass surgery yielded favorable outcomes with low complication and stroke rates. Patients with preoperative stroke require closer follow‐up, and individualized strategies are needed to address persistent functional impairments.

## Graphical abstract

In this European cohort of 276 patients with Moyamoya angiopathy, surgical revascularization, predominantly combined bypass surgery targeting the MCA territory via a mini‐craniotomy, was associated with low perioperative complication rates (6.3%) and stroke rates (2.6%). Long‐term outcomes were favorable, with most patients achieving mRS 0–2, high bypass patency (95.7%), and a low stroke risk of 0.3% per patient‐year. Preoperative stroke and ischemic MRI lesions predicted unfavorable outcomes.

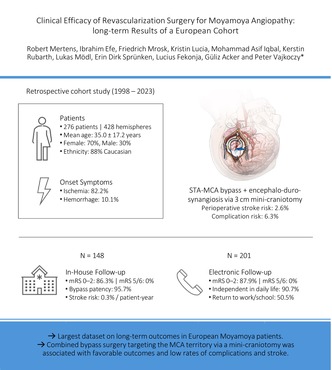

**Abbreviations**
ACA: anterior cerebral artery
ASA: acetylsalicylic acid
CBF: cerebral blood flow
CI: Confidence interval
CTp: computed tomography perfusion
CVRC: cerebrovascular reserve capacity
DSA: digital subtraction angiography
DWI: diffusion‐weighted imaging
ECA: external carotid artery
EC‐IC: extra‐intracranial
EDMAPS: encephalo‐duro‐myo‐arterio‐pericranial‐synangiosis
EDMS: encephalo‐duro‐myo‐synangiosis
EDS: encephalo‐duro‐synangiosis
EGS: encephalo‐galeo‐synangiosis
FU: follow‐up
GEE: generalized estimation equation
ICA: internal carotid artery
ICH: intracerebral hemorrhage
ICU: intensive care unit
IVH: intraventricular hemorrhage
MCA: middle cerebral artery
MMA: Moyamoya angiopathy
MMD: Moyamoya Disease
MMS: Moyamoya syndrome
MRI: magnetic resonance imaging
mRS: Modified Rankin Scale
OR: Odds ratio
PCA: posterior cerebral artery
PET: positron emission tomography
rCBF: regional cerebral blood flow
SAH: subarachnoid hemorrhage
SD: standard deviation
SE: standard error
SPECT: single‐photon emission computed tomography
STA: superficial temporal artery
TIA: transient ischemic attack
uMMD: unilateral Moyamoya Disease
uMMS: unilateral Moyamoya syndrome

## 1 Introduction

Moyamoya Disease (MMD) is a cerebral angiopathy of unknown etiology, characterized by progressive steno‐occlusion of the terminal internal carotid arteries (ICA) with compensatory formation of collaterals resembling a ‘puff of smoke’ (Japanese: Moyamoya) on cerebral angiography [1]. The vascular alterations of the idiopathic MMD and secondary Moyamoya Syndrome (MMS) are summarized as Moyamoya angiopathy (MMA) and lead to similar symptoms, resulting from the stenosis of the ICA (ischemia) and the fragility of compensatory collaterals (hemorrhage). Since its first description by Takeuchi and Shimizu in Japan [2], MMD has been observed worldwide, with highest incidence in East Asian populations (about 1 in 100.000) and a tenfold lower rate in Western countries. Several studies have demonstrated significant interethnic differences between Asian and Caucasian patients, not only in disease epidemiology [3, 4, 5] but also in clinical presentation [6, 7]. Since no causal therapy is available, the only established treatment to restore the cerebral blood flow (CBF) is surgical revascularization [8]. Most series reporting outcomes after surgical revascularization have been published from East Asian countries. In Caucasian patients, available data are limited to small outcome series. Only three studies reported follow‐up (FU) longer than 5 years after direct or combined bypass surgery in adults, and only one of these was conducted in Europe [9, 10, 11]. This study aimed to evaluate long‐term clinical and radiological outcomes in European MMA patients treated with surgical revascularization, focusing on standardized combined bypass surgery via mini‐craniotomy.

## 2 Methods

### 2.1 Study Design

This study was conducted in accordance with the Declaration of Helsinki and approved by the ethics committee of Charité—Universitätsmedizin Berlin (EA2/178/18 and EA2/225/20). This study analyzed retrospectively and prospectively collected data of patients with MMA who underwent surgical revascularization by the senior author at the Department of Neurosurgery, University Medical Center Mannheim (1998–2007), and at Charité—Universitätsmedizin Berlin (2008–2023). MMD and MMS were diagnosed following the 2021 guidelines by the Research Committee on Moyamoya Disease of Japan [12]. This study is reported in accordance with the STROBE guidelines for observational studies.

### 2.2 Patient Characteristics

Age was defined as the age at first surgery. Patients < 18 years were classified as pediatric. Initial symptoms were categorized as ischemic or hemorrhagic. Ischemic symptoms included transient ischemic attacks (TIA) and ischemic strokes, while hemorrhagic symptoms referred to subarachnoid (SAH), intracerebral (ICH), or intraventricular hemorrhage (IVH). Other symptoms such as epilepsy, headache, or cognitive impairment were only considered onset symptoms if neither ischemia nor hemorrhage was present.

### 2.3 Radiological Imaging

Preoperative radiological workup included magnetic resonance imaging (MRI), digital subtraction angiography (DSA), and regional cerebral blood flow (rCBF) measurement for the assessment of the cerebrovascular reserve capacity (CVRC). Each hemisphere was analyzed separately. Ischemic strokes were identified on T2/FLAIR and DWI MRI sequences and classified as watershed, territorial, lacunar, or multifocal [6]. The Suzuki Grade of each hemisphere was determined on preoperative DSA [13]. Assessment of cerebrovascular hemodynamics was performed using acetazolamide‐stimulated ([^99m^Tc]Tc‐HMPAO) single‐photon emission computed tomography (SPECT) or [^15^O]H_2_O positron emission tomography (PET). The qualitative assessment of the CVRC was performed as previously described [14, 15, 16]. Furthermore, each hemisphere was categorized according to the Berlin Grading System, an additive score based on MRI, CVRC, and collateral patterns on DSA to stratify ischemic risk [17].

### 2.4 Surgical Strategy and Postoperative Complications

**FIGURE 1:**
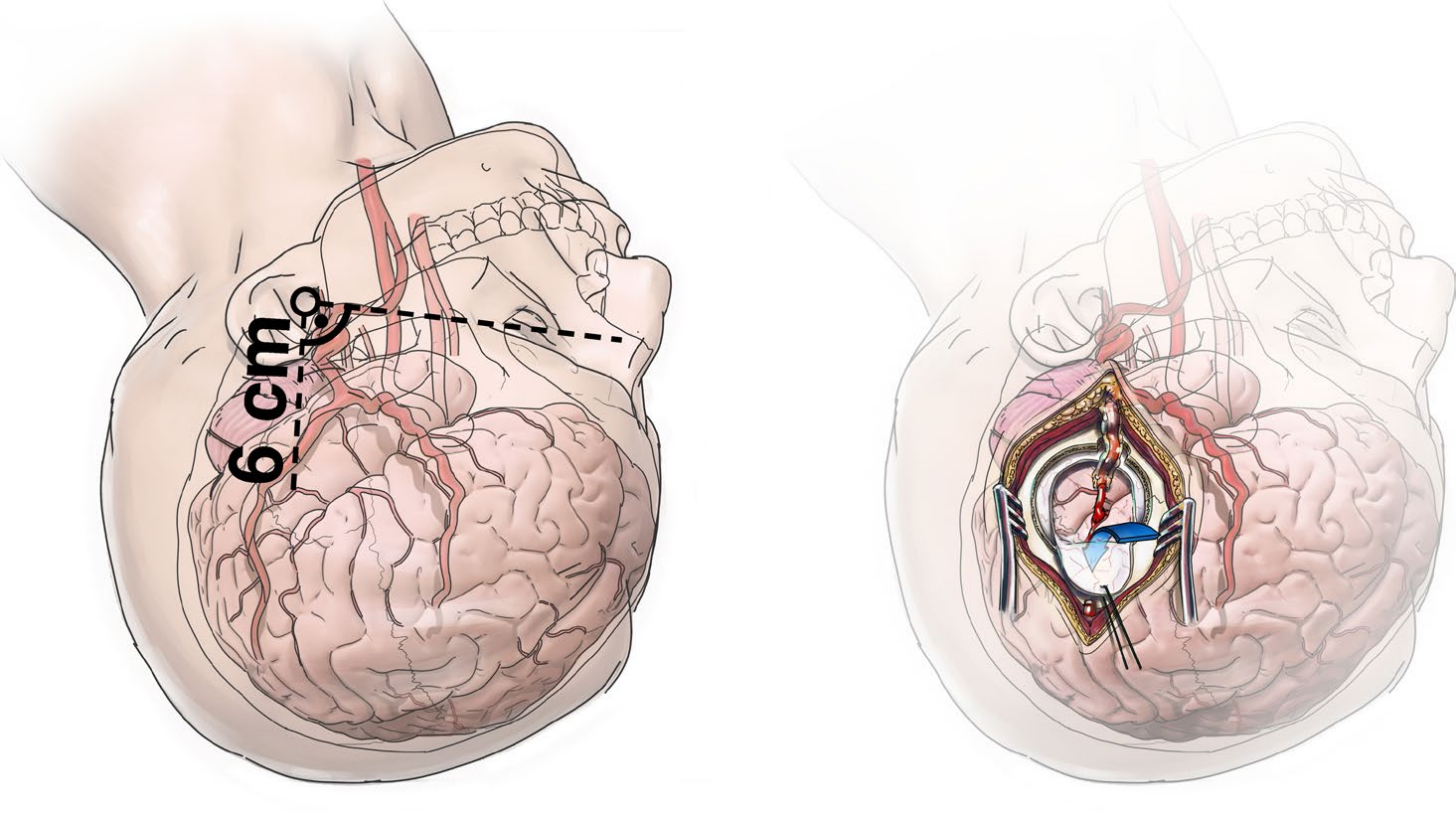
Illustration of the standardized mini‐approach for combined bypass surgery targeting the MCA territory. A specially designed template was used to identify the end of the Sylvian fissure as the optimal cortical target point to reliably find suitable MCA recipient vessels for the direct bypass anastomosis. The handle of the template was positioned in the external auditory canal, with the horizontal reference line aligned with the lateral canthus of the eye. From the external auditory canal, a perpendicular 6 cm line extends, terminating at the target point. At this location, a small craniotomy with a diameter of 3 cm was performed. Following the direct STA‐M4‐MCA bypass, the dural flap was inverted and laid on the cortex to facilitate indirect revascularization (EDS, blue arrow). Further closure was performed, with the STA exiting through the burr hole. EDS—encephalo‐duro‐synangiosis; MCA—middle cerebral artery; STA—superficial temporal artery.

Surgical decision‐making was based on clinical symptoms, MRI lesions, and impaired CVRC. In ischemic MMA, revascularization was performed in the presence of clinical symptoms and/or corresponding MRI lesions or impaired CVRC. In asymptomatic hemispheres, surgery was indicated only if reduced CVRC and corresponding silent ischemic lesions were present. In hemorrhagic MMA, revascularization was performed following intracranial hemorrhage, particularly in patients with hemodynamic impairment and high‐risk collateral patterns. In patients with bilateral disease, the symptomatic hemisphere was treated first, followed by the contralateral hemisphere after 3 months if indicated. If both hemispheres were symptomatic, surgical priority was determined according to the Berlin Grading System. Regarding surgical timing, revascularization was generally performed at least 3 months after an acute cerebrovascular event. Direct revascularization procedures included superficial temporal artery (STA) to M4 middle cerebral artery (MCA) bypass surgery. If no STA was available or the STA‐MCA bypass failed, a radial artery graft (intermediate‐flow bypass) or saphenous vein graft (high‐flow bypass) was used as a rescue strategy in adults. Indirect techniques comprised encephalo‐duro‐synangiosis [18] (EDS) or encephalo‐duro‐myo‐synangiosis (EDMS). In combined procedures, a direct bypass was combined with indirect revascularization techniques. Importantly, adult patients were treated using a standardized mini‐approach with combined revascularization targeting the MCA territory (Figure 1). This strategy involves a small craniotomy of 3 cm diameter, placed with a template to reliably identify the optimal cortical target point and ensure access to suitable MCA recipient vessels for direct anastomosis at the end of the Sylvian fissure while minimizing surgical exposure [19], combined with EDS. In pediatric patients, STA‐MCA bypass was combined with EDMS using a larger fronto‐temporal craniotomy in symptomatic hemispheres, whereas asymptomatic hemispheres were treated using the combined mini‐approach if indicated. Perioperative management included medical treatment with acetylsalicylic acid (ASA) and invasive blood pressure monitoring in the intensive care unit (ICU). CT angiography was performed 24 h postoperatively to confirm bypass patency. Complications were defined as surgery‐ or disease‐related events occurring within 30 days after surgery.

### 2.5 Clinical and Radiological Follow‐Up Assessment

In‐house FU included clinical examination at 3 and 6 months, and clinical plus MRI/DSA assessments 1 year after surgery. Thereafter, evaluations were repeated every 3–5 years. Bypass patency rates refer to the direct bypass component. Functional outcome was assessed using the modified Rankin Scale (mRS) [20]. Furthermore, a three‐grade classification system was applied to quantify the development of preoperative symptoms after surgery: favorable outcome (symptom improvement), moderate outcome (stable symptoms), and worse outcome (worsening or new symptoms). For statistical analysis, moderate and worse outcomes were grouped as unfavorable outcome. Given the rarity of the disease and the resulting wide geographic catchment area, remote FU was implemented to improve coverage. Accordingly, all patients, including those unable to attend onsite FU, were evaluated via telephone or email using a standardized questionnaire (Data S1). Reported ischemic strokes, TIAs, hemorrhages, and epilepsy required clinical confirmation. Relatives were contacted if patients could not be reached. Importantly, the FU modalities partly overlapped, as some patients underwent both in‐house and electronic questionnaire‐based FU. Long‐term FU was defined as ≥ 5 years. For overall and long‐term outcome analyses, the latest available FU after surgery was used, and outcomes were compared between MMD and MMS.

### 2.6 Statistical Analysis

Statistical analysis was performed using GraphPad Prism 10.1.1 and R 4.3.0, with a significance level of α = 0.05. Categorical variables were reported as proportions and continuous variables as means with standard deviations. Group comparison used two ‐ sample proportions tests, χ^2^ tests or Fisher's exact tests for nominal variables as appropriate and Mann – Whitney U tests for ordinal variables. Normality was assessed using the Shapiro – Wilk test, with *t*‐tests or Mann – Whitney U tests performed for continuous variables. Due to the exploratory character of the study, no adjustment for multiplicity was conducted. To identify risk factors for unfavorable clinical outcome in the overall in‐house FU group, univariate and multiple logistic regressions were performed, including age, gender, hemisphere (left/right), prior stroke (clinical and MRI‐confirmed), hemorrhage, TIA, silent ischemic lesions on MRI, CVRC, Berlin Grade, Suzuki stage, and time since surgery. Associations between predictors and outcome were evaluated using Wald χ^2^ tests.

## 3 Results

### 3.1 Patient Characteristics

**FIGURE 2:**
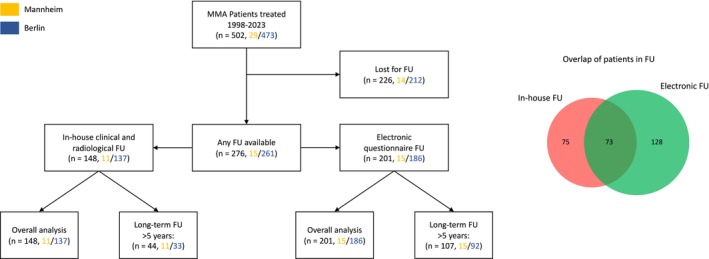
Flow chart demonstrating the inclusion process of MMA patients for FU analysis. A total of 502 patients were treated at the Department of Neurosurgery at the University Medical Center Mannheim (yellow) and at the Charité—Universitätsmedizin Berlin (blue) between 1998 and 2023. FU data was available for a total of 276 patients with 509 affected and 428 surgically treated hemispheres. Among these, 148 patients underwent in‐house clinical and radiological FU, and 201 patients were evaluated by electronic questionnaire, with an overlap of 73 patients included in both groups. FU—Follow‐up; MMA—Moyamoya angiopathy.

Between 1998 and 2023, 502 patients underwent surgical treatment. FU data were available for a total of 276 patients, with 509 affected and 428 surgically treated hemispheres (Table 1). Among these, 148 patients underwent in‐house FU, and 201 were evaluated via electronic questionnaire, with an overlap of 73 patients included in both groups (Figure 2). There were no statistically significant differences between these FU groups. Of the 276 patients with available FU, the majority were diagnosed with bilateral MMD (65.6%), followed by bilateral MMS (18.8%). The mean age at first surgery was 35.0 ± 17.2 years (range 1–71 years), with a female‐to‐male ratio of 2.3:1. The age difference between female and male patients was not significant (*p* = 0.87). Ethnic origin was Caucasian (88.0%), Middle Eastern (6.2%), Asian (4.7%), and African (1.1%). Ischemic events were the most common onset symptom (82.2%), followed by hemorrhage (10.1%). Children were significantly more likely to present with MMS (29.6% vs. 16.2%; *p* = 0.02). Ischemic alterations on preoperative MRI were found in 44.0% of hemispheres, with watershed ischemia being the most common pattern (24.2%; Table 2A). The majority of hemispheres were classified as Suzuki Grades 2–4 and Berlin Grade 3. Preoperative CVRC assessment was performed in 221 of 276 patients (80.1%), and reduced CVRC was observed in 334 of 428 surgically treated hemispheres (78.0%).

**TABLE 1:** Overall patient characteristics.

	All patients (*n* = 276)	Adult patients (*n* = 222, 80.4%)	Pediatric patients (*n* = 54, 19.6%)	*p* ^§^
Age in years (mean ± SD, range)	35.0 ± 17.2 (1–71)	41.7 ± 11.9 (18–71)	8.6 ± 5.2 (1–17)	**< 0.0001** ^**†**^
Gender (M:F)				
Male	84 (30.4%)	64 (28.8%)	20 (37.0%)	0.24
Female	192 (69.6%)	158 (71.2%)	34 (63.0%)	0.24
M:F Ratio	1:2.3	1:2.5	1:1.7	
Diagnosis (%)				
MMD	181 (65.6%)	152 (68.5%)	29 (53.7%)	**0.04**
Unilateral MMD	35 (12.7%)	29 (13.1%)	6 (11.1%)	0.7^‡^
MMS	52 (18.8%)	36 (16.2%)	16 (29.6%)	**0.02**
Unilateral MMS	8 (2.9%)	5 (2.3%)	3 (5.6%)	0.19^‡^
Symptoms at onset (%)				
Ischemic Symptoms	227 (82.2%)	183 (82.4%)	44 (81.5%)	0.87
TIA	139 (50.4%)	112 (50.5%)	27 (50.0%)	
Stroke	88 (31.9%)	71 (32.0%)	17 (31.5%)	
Hemorrhage	28 (10.1%)	25 (11.3%)	3 (5.6%)	0.21^‡^
Other Symptoms	19 (6.9%)	13 (5.9%)	6 (11.1%)	0.17
Epilepsy	4 (1.5%)	2 (0.9%)	2 (3.7%)	
Headache	11 (4.0%)	9 (4.1%)	2 (3.7%)	
Cognitive Impairment	4 (1.5%)	2 (0.9%)	2 (3.7%)	
Asymptomatic	2 (0.7%)	1 (0.5%)	1 (1.9%)	0.28^‡^
Ethnicity				
Caucasian	243 (88.0%)	200 (90.1%)	43 (79.6%)	**0.03**
Asian	13 (4.7%)	12 (5.4%)	1 (1.9%)	0.27^‡^
Middle Eastern	17 (6.2%)	8 (3.6%)	9 (16.7%)	**< 0.001** ^**‡**^
African	3 (1.1%)	2 (0.9%)	1 (1.9%)	0.55^‡^

Abbreviations: MMD; Moyamoya Disease; MMS, Moyamoya Syndrome; SD, Standard Deviation; TIA, Transient Ischemic Attack.

^§^ *p*‐values were obtained using a two‐sample proportions test.

^†^ Welch's *t*‐test was performed.

^‡^ Sample size was insufficient to calculate reliable *p*‐value.

**TABLE 2:** Preoperative radiographic findings of 509 affected hemispheres (A) and surgical strategy in 428 treated hemispheres (B).

A: Preoperative radiographic findings of 509 affected MMA hemispheres.	
	Affected hemispheres (*n* = 509 in 276 patients)
Suzuki‐Grading (*n*, %)	
Grade 1	47/426 (11.0%)
Grade 2	110/426 (25.8%)
Grade 3	114/426 (26.8%)
Grade 4	99/426 (23.2%)
Grade 5	40/426 (9.4%)
Grade 6	16/426 (3.8%)
Ischemic alterations on MRI (*n*, %)	
Any ischemic alteration in MRI	224/509 (44.0%)
Watershed	123/509 (24.2%)
Territorial	27/509 (5.3%)
Lacunar	19/509 (3.7%)
Mixed	55/509 (10.8%)
Berlin Grading (*n*, %)	
Grade 1	39/329 (11.9%)
Grade 2	100/329 (30.4%)
Grade 3	190/329 (57.8%)

B: Surgical strategy in 428 treated MMA hemispheres.		
	Adult hemispheres (*n* = 341, 79.7%)	Pediatric hemispheres (*n* = 87, 20.3%)
Direct Bypass	44 (12.9%)	8 (9.2%)
STA‐M4‐MCA‐Bypass	41 (12.0%)	8 (9.2%)
Intermediate‐Flow‐Bypass	2 (0.6%)	0 (0%)
High‐Flow‐Bypass	1 (0.3%)	0 (0%)
Indirect Bypass	3 (0.9%)	8 (9.2%)
Combined Bypass	294 (86.2%)	71 (81.6%)
STA‐MCA + EDS (Standardized mini‐approach)^§^	276 (80.9%)	30 (34.5%)
STA‐MCA + EDMS	18 (5.3%)	41 (47.1%)

Abbreviations: EDMS, Encephalo‐Duro‐Myo‐Synangiosis; EDS, Encephalo‐Duro‐Synangiosis; MCA, Middle Cerebral Artery; MMA, Moyamoya Angiopathy; MRI, Magnetic Resonance Imaging; STA, Superficial Temporal Artery.

^§^ For standardized combined revascularization, a small craniotomy of 3 cm diameter is placed with a standardized template. Following the direct STA‐M4‐MCA bypass, the dural flap is inverted and laid on top of the cortex for indirect revascularization (EDS).

### 3.2 Surgical Strategy and Safety

A total of 428 hemispheres from 276 patients were treated surgically (Table 2B). In adult patients, combined bypass surgery using the standardized mini‐approach was the treatment of choice (80.9%). In pediatric patients, symptomatic hemispheres were treated with STA‐MCA bypass combined with EDMS (47.1%) and asymptomatic hemispheres were treated using the standardized mini‐approach (34.5%). In a minority of cases, the planned indirect component (EDS or EDMS) was not performed due to intraoperative technical considerations, such as unintended dural compromise during craniotomy or anatomical constraints precluding safe dural inversion or adequate placement of indirect tissue. If the STA was too fragile for direct bypass in children, an indirect technique was used alone (9.2%). Postoperative complications occurred in 27 of 428 surgeries (6.3%). These included ischemic stroke (*n* = 11) with a perioperative stroke risk of 2.6%, wound healing disorders requiring surgery (*n* = 6, 1.4%), seizures (*n* = 4, 0.9%), subdural hematomas requiring evacuation (*n* = 3, 0.7%), and hyperperfusion syndrome (*n* = 3, 0.7%).

### 3.3 In‐House Follow‐Up

Regarding the overall outcome analysis of 148 patients with 242 treated hemispheres who underwent in‐house FU (Table 3), the mean time to the latest available FU was 41.2 ± 35.5 months (range 3–131 months). A total of 6/148 patients (4.1%) developed new ischemic radiological lesions, with no cases of hemorrhage. Among these, 3/148 patients (2.0%) presented with new neurological symptoms. The estimated risk of new radiological and clinical stroke was 1.2% and 0.6% per patient‐year, respectively. New ischemic radiological lesions occurred equally in pediatric and adult patients (2/32 vs. 4/116; *p* = 0.48). Angiographic evaluation at the latest FU was available for 189 hemispheres and showed a bypass patency rate of 96.3%. Regarding the development of preoperative symptoms, 85.1% of patients had a favorable outcome, 12.8% a moderate outcome, and 2.0% a worse outcome. Of the 148 patients, 44 (29.7%) were included in the long‐term FU group, with a mean time to the latest available FU of 88.6 ± 21.0 months (range 61–131 months). In this group, new ischemic radiological lesions were detected in 2 patients (4.5%) on MRI. One of these patients presented with new neurological symptoms, resulting in a long‐term estimated risk of new radiological and clinical stroke of 0.6% and 0.3% per patient‐year, respectively. The bypass patency rate in this subgroup was 95.7%, with symptom improvement in 86.4% of patients and worsening in 2.3% of patients. Regarding the functional outcome according to the mRS, scores of 5 and 6 were not observed, and 91.9% of the overall group and 86.3% of the long‐term group had an mRS score of 0–2.

**TABLE 3:** Clinical and radiological outcomes of 148 patients who underwent in‐house FU. A total of 44 patients underwent FU for more than 60 months / 5 years (long‐term subgroup). Ischemic and hemorrhagic lesions as well as clinical outcome were evaluated per patient. Bypass patency was only included if angiography was available at latest FU and was evaluated per treated hemisphere.

	Overall (*n* = 148)	Long‐term (> 5 years) (*n* = 44)
Mean time until latest available FU		
Months ± SD (range)	41.2 ± 35.5 (3–131)	88.6 ± 21.0 (61–131)
Years ± SD (range)	3.4 ± 3.0 (0.25–10.9)	7.4 ± 1.8 (5.1–10.9)
New radiological ischemic lesions (symptomatic lesions)^§^	6/148, 4.1% (3)	2/44, 4.5% (1)
Risk for new radiological stroke/patient‐year	1.2%	0.6%
Risk for new clinical stroke/patient‐year	0.6%	0.3%
New radiological hemorrhagic lesions (symptomatic lesions)^§^	0 (0)	0 (0)
Occlusion of direct bypass (%)^†^	7/189 (3.7%)	2/46 (4.3%)
Bypass occlusion in patients with new ischemic lesions (%)	4/6 (66.7%)	0/2 (0%)
Change in preoperative symptoms (%)^§^		
Favorable: symptom improvement	126/148 (85.1%)	38/44 (86.4%)
Moderate: stable symptoms	19/148 (12.8%)	5/44 (11.4%)
Worse: worsening/new symptoms	3/148 (2.0%)	1/44 (2.3%)
Modified Rankin Scale (mRS)^§^		
0	91/148 (61.5%)	25/44 (56.8%)
1	25/148 (16.9%)	7/44 (15.9%)
2	20/148 (13.5%)	6/44 (13.6%)
3	7/148 (4.7%)	3/44 (6.8%)
4	5/148 (3.4%)	3/44 (6.8%)
5	0/148 (0%)	0/44 (0%)
6	0/148 (0%)	0/44 (0%)

Abbreviations: FU, Follow‐Up; MRS, Modified Rankin Scale; SD, Standard Deviation.

^§^ Evaluated per patient.

^†^ Evaluated per hemisphere if available.

### 3.4 Risk Factors for Unfavorable Clinical Outcome

In the univariate analysis, preoperative ischemic stroke (OR: 0.212%; 95% CI: 0.082, 0.550; *p* = 0.001) and silent ischemic lesions on MRI (OR: 0.292%; 95% CI: 0.086, 0.987; *p* = 0.048) were statistically significant predictors of an unfavorable outcome, whereas age, gender, hemisphere (left vs. right), preoperative hemorrhage and TIA, CVRC, Berlin Grade, Suzuki Grade, and time since surgery were not (Data S2). In the multiple logistic regression model, preoperative ischemic stroke (OR: 0.318%; 95% CI: 0.122, 0.828; *p* = 0.019) remained a statistically significant predictor of unfavorable outcome, whereas preoperative silent ischemic lesions on MRI were not (OR: 0.638%; 95% CI: 0.245, 1.659; *p* = 0.356). No additional risk predictors were identified in the multiple regression analysis.

### 3.5 Follow‐Up Using Electronic Questionnaires

Among 201 patients with 327 treated hemispheres, the mean overall electronic FU was 75.9 ± 58.8 months (range 5–293; Table 4). In 107 patients with electronic FU ≥ 5 years, the mean was 123.2 ± 47.4 months (range 60–293). Regarding the functional outcome according to the mRS, scores of 5 and 6 were not observed, and 92.0% of all patients and 87.9% of the long‐term group had an mRS score of 0–2. Approximately 92% of patients in both groups reported an improvement in preoperative symptoms. Independence in daily life was reported by 88.6% of all patients and 90.7% of the long‐term group, while return to pre‐disease occupational or school engagement was achieved by 59.2% and 50.5%, respectively.

**TABLE 4:** Clinical outcomes of 201 patients who underwent FU via electronic questionnaire. A total of 107 patients answered the questionnaire five or more years after the last surgery (long‐term subgroup).

	Overall (*n* = 201)	Long‐term (> 5 years) (*n* = 107)
Mean time until latest available FU		
Months ± SD (range)	75.9 ± 58.8 (5–293)	123.2 ± 47.4 (60–293)
Years ± SD (range)	6.3 ± 4.9 (0.4–24.4)	10.3 ± 4.0 (5.0–24.4)
Modified Rankin Scale (mRS)		
0	51.2% (103)	53.3% (57)
1	31.3% (63)	20.6% (22)
2	9.5% (19)	14.0% (15)
3	5.5% (11)	7.5% (8)
4	2.5% (5)	4.7% (5)
5	0% (0)	0% (0)
6	0% (0)	0% (0)
Overall clinical improvement		
Improvement	92.0% (185)	92.5% (99)
Stable	6.0% (12)	6.5% (7)
Worsening/new symptoms	2.0% (4)	0.9% (1)
Ischemic Stroke^§^		
Pts. who experienced stroke preoperatively, number of pts. experiencing stroke post‐surgery	5.1% (4/79)	4.4% (2/45)
In pts. with no stroke preoperatively, number of pts. with new stroke post‐surgery	1.6% (2/122)	3.2% (2/62)
Transient Ischemic Attacks (TIA)^§^		
In pts. with TIAs preoperatively, development of TIAs post‐surgery (n)	111	56
Improvement	91.9% (102)	92.9% (52)
Stable	5.4% (6)	5.4% (3)
Worsening	2.7% (3)	1.8% (1)
In pts. with no TIAs preoperatively, number of pts. with new TIAs post‐surgery	5.6% (5/90)	7.8% (4/51)
Intracranial hemorrhage^§^		
In pts. with intracranial hemorrhage preoperatively, number of pts. with new intracranial hemorrhage post‐surgery	0% (0/19)	0% (0/11)
In pts. with no intracranial hemorrhage preoperatively, number of pts. with new intracranial hemorrhage post‐surgery	0% (0/182)	0% (0/96)
Headache		
In pts. with headache preoperatively, development of headache post‐surgery (n)	67	37
Improvement	71.6% (48)	70.3% (26)
Stable	23.9% (16)	27.0% (10)
Worsening	4.5% (3)	2.7% (1)
In pts. with no headache preoperatively, number of pts. with new headache post‐surgery (no surgical pain)	3.7% (5/134)	2.9% (2/70)
Epileptic seizures^§^		
In pts. with epileptic seizures preoperatively, development of epileptic seizures post‐surgery (n)	16	7
Improvement	81.3% (13)	71.4% (5)
Stable	18.8% (3)	28.6% (2)
Worsening	0% (0)	0% (0)
In pts. with no epileptic seizures preoperatively, number of pts. with new epileptic seizures post‐surgery	2.2% (4/185)	2.0% (2/100)
Patient independence	88.6% (178)	90.7% (97)
Return to work/school as before disease onset	59.2% (119)	50.5% (54)

Abbreviations: FU; follow‐up; MRS; Modified Rankin Scale; PTS; Patients; SD; Standard Deviation; TIA; Transient Ischemic attack.

^§^ Clinically confirmed.

### 3.6 Comparison of Moyamoya Disease and Moyamoya Syndrome

MMD and MMS patients were compared regarding demographics and outcomes based on both in‐house and electronic overall FU data. MMS patients were significantly younger (30.7 ± 19.1 years; range 2–69) than MMD patients (36.4 ± 16.4 years; range 1–71; *p* = 0.049). The median [interquartile range] mRS score in the in‐house FU group was 0 [0–1] for both MMD patients (*n* = 117) and MMS patients (*n* = 31). In the electronic FU group, the median mRS score was 0 [0–1] for MMD patients (*n* = 156) and 1 [0–2] for MMS patients (*n* = 45). MRS scores did not differ between MMD and MMS in the in‐house FU group (*p* = 0.716), whereas significantly worse scores were observed in MMS in the electronic FU group (*p* = 0.006). Furthermore, MMD patients were more likely to be independent in daily life (92.3% vs. 75.6%; *p* = 0.001).

## 4 Discussion

This study provides long‐term outcome data from one of the largest European cohorts of predominantly Caucasian MMA patients, comprising 276 individuals with 428 revascularized hemispheres. Most patients underwent a standardized mini‐approach targeting the MCA territory, demonstrating that surgical revascularization is safe and effective in this population. Perioperative morbidity was low, with a 6.3% overall complication rate and a 2.6% perioperative stroke risk. Long‐term outcomes were highly favorable, with bypass patency of 95.7% and annual risks of new radiological and clinical stroke of 0.6% and 0.3% per patient‐year, respectively. Functionally, 86.3%–87.9% of patients achieved mRS 0–2, and severe disability or death (mRS ≥ 5) was not observed. Moreover, more than 90% reported improvement in preoperative symptoms and independence in daily life, and approximately half successfully returned to work or school, highlighting the durable benefit of surgical revascularization and its relevance for long‐term functional recovery.

### 4.1 Patient Characteristics

Demographic and clinical data of our European MMA cohort were comparable to other Caucasian studies [5, 9, 10, 21, 22]. The percentage of Caucasian patients in our study was notably high (88.0%, compared to 54% in the study by Teo et al. [9]), resulting in a greater homogeneity regarding ethnicity and genetic background. Ischemic symptoms were the most common onset in our cohort (82.2%), followed by hemorrhage (10.1%), consistent with Teo et al. (82% vs. 13%) [9], Birkeland et al. (47% vs. 23%) [5] and Krämer et al. (71.5%/82% vs. 9.5%) [21, 22]. Teo et al. reported headache and seizures in 46% and 12% of patients [9], compared to 4.0% and 1.5% in our study. However, comparison is limited by differing definitions, as these symptoms were only counted as onset in our study if neither ischemia nor hemorrhage were present, which resulted in an underestimation of their true frequency. Teo et al. found significantly more hemorrhagic presentations in adults than in children (15.2% vs. 7.3%; *p* = 0.004) [9]. Similarly, our cohort showed a higher rate in adults (11.3% vs. 5.6%), though not statistically significant (*p* = 0.21). In Asian studies, adult patients also present with intracranial hemorrhage more often than pediatric patients [23], but hemorrhage in adult patients was generally more frequent than in European cohorts with frequencies of up to 62.4% [24, 25, 26, 27, 28]. Finally, our cohort showed angiographic features similar to those reported in the European cohort described by Pilgram‐Pastor et al. [29], further supporting the comparability of Caucasian MMA populations.

### 4.2 Surgical Strategy and Safety

Direct and combined bypass procedures have been shown to be superior to indirect revascularization techniques in preventing recurrent strokes and hemorrhages, with the most favorable outcomes reported after combined bypass surgery [30]. Accordingly, current guidelines recommend the preferential use of direct or combined bypass procedures [8, 31]. Our age‐ and symptom‐adapted surgical strategy reflects both technical and pathophysiological considerations. In pediatric patients, disturbances in cerebral hemodynamics are more pronounced than in adults [32], and direct anastomosis can be technically more demanding due to smaller vessel caliber. At the same time, the higher angiogenic potential in children favors the additional use of indirect revascularization strategies [1, 32]. Therefore, a broader indirect component was reserved for symptomatic pediatric hemispheres, which were treated with STA‐MCA bypass combined with EDMS. In contrast, asymptomatic pediatric hemispheres and adult hemispheres were managed using the standardized combined mini‐approach, as this strategy provides effective revascularization while limiting surgical exposure. Adding EDS to a direct bypass by inverting the dura is a technically simple adjunct and provides an additional source of vessel ingrowth into the cortex. Although the maturation of EDS typically lags behind that of the STA‐MCA bypass, it may serve as a complementary and additive revascularization source when combined with a direct bypass [18]. In our study, combined bypass using a standardized mini‐approach was performed in 80.9% of adult patients. This approach is less invasive than for example STA‐MCA anastomosis and encephalo‐duro‐myo‐arterio‐pericranial‐synangiosis (EDMAPS) performed via a large craniotomy, as practiced in Japan [33]. Supporting this statement, our technique was associated with fewer perioperative complications (6.3% vs. 13.5%) and a lower risk of hyperperfusion (0.7% vs. 4.3%), likely due to a smaller mismatch between preoperative chronic hypoperfusion and rapid postoperative increase in CBF. Kuroda et al. reported a perioperative stroke risk of 3.5% per procedure [33]. With the mini‐approach, we observed a risk of 2.6%, while Teo et al. reported 3.9% using mainly direct bypass as a stand‐alone technique [9]. Guzman et al. found similar rates, with ischemic stroke and hemorrhage in 1.7% and 1.8%, respectively [34]. Rates of subdural hematoma and wound infection requiring surgery were comparable across studies. In summary, the standardized mini‐approach applied in the vast majority of patients proved to be safe, with a low rate of perioperative complications.

### 4.3 Clinical and Radiological Outcome

Among symptomatic Caucasian patients, annual rates of recurrent stroke and hemorrhage in the natural course of the disease have been reported as high as 13.3% and 1.7%, respectively [35]. Considering these risks and the reported 5‐year stroke recurrence rate of up to 65%–82% under medical therapy [36], the long‐term stroke risk of only 0.3% per patient‐year observed in our in‐house long‐term FU group underscores the benefit of bypass surgery. Teo et al. reported a comparable long‐term ischemic and hemorrhagic stroke risk of 0.6% per patient‐year in 741 patients with a mean FU of 7.3 years after surgery [9]. Guzman et al. reported a mortality rate after surgical revascularization of 2.3% during a mean FU period of 4.9 years [34], and in the study by Teo et al., 40 patients died over a mean FU of 7.3 years [9]. In our study, no patient deaths were recorded during FU. However, loss to FU may have introduced attrition bias, potentially leading to an underestimation of mortality. Despite limited comparability, Kuroda et al. also did not report any deaths and reported a similar long‐term stroke risk of 0.1% per patient‐year after combined STA‐MCA anastomosis and EDMAPS [33]. This comparison is relevant to the ongoing debate on whether MMA patients should be treated with complex, individualized approaches, which often demand multiple bypasses to revascularize different vascular territories, or whether one should aim for a standardized mini‐approach targeting the MCA territory and rely on the leptomeningeal collaterals to the anterior cerebral artery (ACA) and posterior cerebral artery (PCA) territories. Our results may support the latter, demonstrating its clinical effectiveness while reducing surgical complexity and invasiveness. In particular, the low postoperative stroke rate observed in our cohort suggests that additional ACA or PCA bypasses are usually not required in the absence of territory‐specific symptoms or radiological evidence of insufficient collateralization.

Of the 6 patients with new ischemic lesions in the overall cohort, 4 had occlusion of the corresponding bypass, whereas neither of the 2 patients with new ischemic lesions in the long‐term subgroup showed bypass occlusion. Thus, bypass occlusion might not inevitably lead to new ischemia. A recent North American study supports this finding, as only 1 of 17 occluded bypasses was associated with clinical symptoms [37]. This discrepancy between the reduction of stroke risk and stable symptoms despite bypass occlusion remains a subject of future research but may be explained by the additional effect of indirect bypass techniques [38].

At a mean FU of 41 months with a maximum of 11 years for in‐house assessments and 76 months with a maximum of 24 years for remote FU, outcomes were highly favorable. Despite inevitable loss to FU in this large, Europe‐wide cohort, comprehensive outcome data were obtained through standardized electronic questionnaires and direct contact with patients or their relatives. In the overall in‐house FU analysis, 85.1% of patients showed symptom improvement. Only 2.0% worsened in their clinical state, and preoperative ischemic stroke and silent ischemic lesions on MRI were identified as risk factors for an unfavorable outcome. Regarding functional outcome according to the mRS, 91.9% had an mRS score of 0–2, with no scores of 5 or 6. Teo et al. reported mRS scores of 0–1 in 75% of patients [9], similar to our results (72.7% long‐term) and the results of Guzman et al. [34]. Kuroda et al. also did not observe mRS scores of 5 or 6 in their Japanese cohort after surgery and reported scores of 0–1 in 94.6% of patients [33]. In our electronic overall FU, 88.6% reported independency in everyday life. Similarly, 87% of the patients in the study by Teo et al. were self‐caring and 75% were living independently or with family support [9]. Interestingly, 83% of patients in the study by Teo et al. remained employed or in school [9], whereas only 50.5% (long‐term) returned to their pre‐disease level of occupational or academic engagement in our electronic FU. This observation was confirmed in another study [39] and underscores the importance of structured long‐term FU and functional monitoring, even in the presence of favorable neurological outcomes.

## 5 Limitations

Although surgical techniques and perioperative management were consistent throughout the study, advances in equipment and increasing surgical experience may have influenced outcomes. The high rate of loss to FU reflects the long study period, dual‐center inclusion, and patient referral from across Germany and Europe, many of whom returned to local care postoperatively. Baseline characteristics did not differ between included and lost‐to‐FU patients. Nevertheless, loss to FU represents a potential source of attrition bias and may have influenced the reported outcomes. Given the rarity of MMA in non‐Asian countries and the broad catchment area of specialized centers, systematic in‐house FU was often unfeasible. Therefore, electronic questionnaires were employed as a pragmatic approach to assess long‐term outcomes. While this enabled data collection from geographically dispersed patients, reporting bias cannot be excluded due to the patient‐reported nature of the data. In addition, the questionnaire‐based FU lacked standardized neuropsychological testing and detailed validated clinical outcome instruments, thereby limiting the granularity of symptom and outcome assessment. The absence of mRS scores of 5 or 6 likely reflects selection bias, as severely disabled or deceased patients were less likely to respond. To mitigate this, relatives were contacted when patients could not be reached. Overall, the retrospective design and potential underreporting should be considered when interpreting event rates. Nevertheless, this large European series provides valuable insights into the management of MMA and underscores the benefits of surgical revascularization.

## 6 Conclusion

This study provides the largest dataset to date on long‐term outcomes following revascularization surgery for MMA in a European cohort. The standardized combined bypass targeting the MCA territory via a mini‐craniotomy proved to be safe and effective with a low perioperative risk. Most patients experienced favorable outcomes, including good functional status, improvement in preoperative symptoms, independence in daily life, and a low risk of recurrent stroke. Particular attention should be paid to identifying and managing deficits in everyday life, and patients with preoperative ischemic stroke and silent ischemic lesions on MRI require closer monitoring.

## Conflicting Interest

The authors declare no conflicts of interest.

## Author Contributions

**Kerstin Rubarth:** formal analysis, writing – review and editing, visualization. **Robert Mertens:** conceptualization, data curation, formal analysis, writing – original draft, writing – review and editing, methodology, visualization, investigation, validation, project administration, funding acquisition. **Ibrahim Efe:** data curation, writing – review and editing. **Mohammad Asif Iqbal:** data curation, formal analysis, writing – review and editing, visualization, investigation, validation. **Lucius Fekonja:** writing – review and editing, visualization. **Güliz Acker:** conceptualization, writing – review and editing, methodology, supervision, project administration, writing – original draft. **Peter Vajkoczy:** conceptualization, writing – original draft, writing – review and editing, methodology, supervision, project administration, resources. **Lukas Mödl:** formal analysis, writing – review and editing, visualization, investigation, validation. **Kristin Lucia:** data curation, writing – review and editing. **Friedrich Mrosk:** data curation, writing – review and editing. **Erin Dirk Sprünken:** formal analysis, writing – review and editing, visualization, investigation, validation.

## Funding

Robert Mertens received funding from the Moyamoya Freunde und Förderer e.V. Research Scholarship 2022. All other authors report no specific funding related to this work.

## Ethics Statement

All procedures performed in this study involving human participants were in accordance with the ethical standards of the institutional and/or national research committee (ethics committee of the Charité—Universitätsmedizin Berlin, Germany; EA2/178/18 and EA2/225/20) and with the Declaration of Helsinki (1964) and its later amendments or comparable ethical standards.

## Consent

For patients treated before 2020, formal consent for retrospective analysis was waived under ethical approvals EA2/178/18 and EA2/225/20. From 2021 onward, patients were enrolled in a prospective registry study with written informed consent (EA2/225/20).

## Conflicts of Interest

The authors declare no conflicts of interest.

## Supporting information


**Data S1:** Supplement 1: Electronic Follow‐Up Questionnaire.

**Data S2:** Supplement 2: Univariate (A) and multiple (B) logistic regression model of risk predictors for unfavorable clinical outcome in the overall in‐house FU group including 148 patients with 242 treated hemispheres. In order to determine significant risk predictors for unfavorable clinical outcome in the overall in‐house FU group, univariate and multiple logistic regressions were performed. Several variables used for the univariate regression had to be omitted in the multiple regression due perfect separation problems as the number of unfavorable clinical outcomes was too small relative to the sample size. Wald χ2‐tests were conducted to test whether individual parameters were associated with unfavorable clinical outcome. An Odds Ratio < 1 indicates the increased probability of an unfavorable outcome compared to a favorable outcome.

**Data S3:** STROBE Statement—Checklist of items that should be included in reports of cohort studies.

## Data Availability

The data that support the findings of this study are available from the corresponding author upon reasonable request.
