# Supplementary material for: Clinical Efficacy of Revascularization Surgery for Moyamoya Angiopathy: Long‐Term Results of a European Cohort
Source: Eur J Neurol. 2026 Jun 12;33(6):e70664. doi: 10.1111/ene.70664 (PMC13263159; doi:10.1111/ene.70664)
Supplement: Supplementary file 1 — Data S1: Supplement 1: Electronic Follow‐Up Questionnaire. [file ENE-33-e70664-s002.docx]

**Supplement 1: Electronic Follow-Up Questionnaire.**

1. **Overall Symptoms/Deficits**
   - If there were symptoms/deficits before surgery (regardless of their nature or cause), did they improve after surgery?
     - Symptom improvement / stable symptoms / worsening or new symptoms
2. **Clinically confirmed Transient Ischemic Attacks**
   - TIAs before surgery?
     - Yes / No
   - If there were TIAs before surgery, how did they progress after surgery?
     - Improvement / Stable / Worsening
   - New TIAs after surgery?
     - Yes / No
3. **Clinically confirmed Ischemic Stroke**
   - Stroke before surgery?
     - Yes / No
   - New Stroke after surgery?
     - Yes / No
4. **Clinically confirmed Hemorrhagic Stroke / Intracranial hemorrhage**
   - Hemorrhagic stroke before surgery?
     - Yes / No
   - New hemorrhagic stroke after surgery?
     - Yes / No
5. **Clinically confirmed Epilepsy**
   - Epilepsy before surgery?
     - Yes / No
   - If there were epileptic seizures before surgery, how did they progress after surgery?
     - Improvement / Stable / Worsening
   - New Epilepsy after surgery?
     - Yes / No
6. **Headaches (not caused by the surgery)**
   - Headaches before surgery?
     - Yes / No
   - If there were headaches before surgery, how did they progress after surgery?
     - Improvement / Stable / Worsening
   - New headaches?
     - Yes / No
7. **Daily Living and Work/School**
   - Independent in daily activities after surgery?
     - Yes / No
   - Employed / back to school (if <18) after surgery, as before the onset of the disease?
     - Yes / No
