# Supplementary material for: Clinical Efficacy of Revascularization Surgery for Moyamoya Angiopathy: Long‐Term Results of a European Cohort
Source: Eur J Neurol. 2026 Jun 12;33(6):e70664. doi: 10.1111/ene.70664 (PMC13263159; doi:10.1111/ene.70664)
Supplement: Supplementary file 2 — Data S2: Supplement 2: Univariate (A) and multiple (B) logistic regression model of risk predictors for unfavorable clinical outcome in the overall in‐house FU group including 148 patients with 242 treated hemispheres. In order to determine significant risk predictors for unfavorable clinical outcome in the overall in‐house FU group, univariate and multiple logistic regressions were performed. Several variables used for the univariate regression had to be omitted in the multiple regression due perfect separation problems as the number of unfavorable clinical outcomes was too small relative to the sample size. Wald χ2‐tests were conducted to test whether individual parameters were associated with unfavorable clinical outcome. An Odds Ratio < 1 indicates the increased probability of an unfavorable outcome compared to a favorable outcome. [file ENE-33-e70664-s001.docx]

**Supplement 2: Univariate (A) and multiple (B) logistic regression model of risk predictors for unfavorable clinical outcome in the overall in-house FU group including 148 patients with 242 treated hemispheres.** In order to determine significant risk predictors for unfavorable clinical outcome in the overall in-house FU group, univariate and multiple logistic regressions were performed. Several variables used for the univariate regression had to be omitted in the multiple regression due perfect separation problems as the number of unfavorable clinical outcomes was too small relative to the sample size. Wald χ2-tests were conducted to test whether individual parameters were associated with unfavorable clinical outcome. An Odds Ratio <1 indicates the increased probability of an unfavorable outcome compared to a favorable outcome.

**A: Univariate logistic regression model.**

| Predictor | Hemispheres | Patients | Coefficient  (Odds Ratio) | 95% CI | | p-value |
| --- | --- | --- | --- | --- | --- | --- |
|  |  |  |  | Lower Limit | Upper Limit |  |
| Age | 242 | 148 | 0.997 | 0.971 | 1.022 | 0.797 |
| Gender | 242 | 148 | 1.181 | 0.392 | 3.557 | 0.768 |
| Hemisphere (L/R) | 242 | 148 | 1.100 | 0.547 | 2.210 | 0.790 |
| Ischemic stroke | 242 | 148 | 0.212 | 0.082 | 0.550 | **0.001** |
| Hemorrhage | 242 | 148 | 3.111 | 0.414 | 23.383 | 0.270 |
| TIA | 242 | 148 | 1.508 | 0.606 | 3.758 | 0.377 |
| Silent ischemic lesions on MRI | 242 | 148 | 0.292 | 0.086 | 0.987 | **0.048** |
| CVRC | 184 | 115 | 0.828 | 0.448 | 1.528 | 0.545 |
| Berlin Grade | 184 | 115 | 1.195 | 0.657 | 2.173 | 0.559 |
| Suzuki | 242 | 148 | 0.766 | 0.524 | 1.122 | 0.171 |
| Time since surgery | 242 | 148 | 1.000 | 1.000 | 1.001 | 0.568 |

**B: Multiple logistic regression model.** 184 hemispheres of 115 patients were included. Score test = 0.377.

| Predictor | Coefficient  (Odds Ratio) | 95% CI | | p-value |
| --- | --- | --- | --- | --- |
|  |  | Lower Limit | Upper Limit |  |
| (Intercept) | 0.311 | 0.021 | 4.554 | 0.393 |
| Age | 0.987 | 0.947 | 1.028 | 0.540 |
| Gender | 0.961 | 0.294 | 3.136 | 0.947 |
| Hemisphere (L/R) | 0.934 | 0.476 | 1.835 | 0.844 |
| Ischemic stroke | 0.318 | 0.122 | 0.828 | **0.019** |
| TIA | 1.855 | 0.648 | 5.317 | 0.250 |
| Silent ischemic lesions on MRI | 0.638 | 0.245 | 1.659 | 0.356 |
| CVRC | 0.973 | 0.385 | 2.460 | 0.954 |
| Time since surgery | 1.000 | 1.000 | 1.001 | 0.461 |

CI – Confidence interval; CVRC – cerebrovascular reserve capacity; FU – follow-up; MRI – magnetic resonance imaging; TIA – transient ischemic attack.
